# Ovarian Transcriptomic Analysis of Ninghai Indigenous Chickens at Different Egg-Laying Periods

**DOI:** 10.3390/genes13040595

**Published:** 2022-03-27

**Authors:** Xuan Huang, Wei Zhou, Haiyue Cao, Haiyang Zhang, Xin Xiang, Zhaozheng Yin

**Affiliations:** 1 Zijingang Campus, Animal Science College, Zhejiang University, Hangzhou 310058, China; 22017085@zju.edu.cn (X.H.); 21917002@zju.edu.cn (W.Z.); 22017062@zju.du.cn (H.Z.); 22117003@zju.edu.cn (X.X.); 2 College of Animal Science and Technology, Jiangsu Agri-Animal Husbandry Vocational College, Taizhou 225300, China; hssmycao@163.com

**Keywords:** Ninghai chicken, RNA-seq, egg production, ovary

## Abstract

Egg production is an essential indicator of poultry fertility. The ovary is a crucial organ involved in egg production; however, little is known about the key genes and signaling pathways involved in the whole egg-laying cycle of hens. In order to explore the mechanism of egg production at different stages of the egg-laying process, ovarian tissues from four chickens were randomly selected for transcriptome analysis at each of the three ages (145 d, 204 d, and 300 d in the early, peak, and late stages of egg laying). A total of 12 gene libraries were constructed, and a total of 8433 differential genes were identified from NH145d vs. NH204d, NH145d vs. NH300d and NH300d vs. NH204d (Ninghai 145-day-old, Ninghai 204-day-old, and Ninghai 300-day-old), with 1176, 1653 and 1868 up-regulated genes, and 621, 1955 and 1160 down-regulated genes, respectively. In each of the two comparison groups, 73, 1004, and 1030 differentially expressed genes were found to be co-expressed. We analyzed the differentially expressed genes and predicted nine genes involved in egg production regulation, including *LRP8, BMP6, ZP4, COL4A1, VCAN, INHBA, LOX, PTX3*, and *IHH*, as well as several essential egg production pathways, such as regulation adhesion molecules (CAMs), calcium signaling pathways, neuroactive ligand–receptor interaction, and cytokine–cytokine receptor interaction. Transcriptional analysis of the chicken ovary during different phases of egg-lay will provide a useful molecular basis for study of the development of the egg-laying ovary.

## 1. Introduction

Egg production is an important indicator of the reproductive performance of poultry and an economically important trait. Eggs are an economical source of high-quality protein and important vitamins and minerals that play a vital role in the human diet [1]. Eggs are consumed worldwide, and their consumption will continue to grow in the future due to their low price and nutritional benefits [2]. The ovary is a vital component of the female reproductive system. Its main roles are to produce eggs and regulate oocyte secretion by releasing sex hormones for ovulation, and the secretion of hormones essential for regulating oocyte development, follicle maturation, and the ovulation process [3]. Eggs develop from follicles, and the process of egg formation in the ovary is a complicated process that is influenced by environmental and nutritional factors [4], as well as by the hypothalamic-pituitary-gonadal (HPG) axis, which produces specific neuropeptides or hormones that stimulate oocyte maturation and ovulation [5]. Furthermore, ovarian follicular development also involves various endocrine, autocrine, and paracrine factors, which control the proliferation and differentiation of oocytes, granulosa cells, and thecal cells [6]. RNA sequencing (RNA-seq) is a transcriptome analysis approach that provides information at the single nucleotide level regarding the complete biological transcript [7]. It utilizes deep sequencing techniques to address biological questions and allows the detection of differences in gene expression profiles between individuals of different developmental stages at the transcriptional level of whole-genome sequencing, facilitating the study of gene function. Transcriptome sequencing has been conducted in numerous studies to identify ovarian genes in livestock such as pigs [8], cattle [9], sheep [10] and goats [11]. Zou et al. identified 1027 differentially expressed genes in the ovaries of high- and low-yielding Leizhou black ducks, of which 495 genes were up-regulated and 532 genes were down-regulated, and found fifty genes that were related to reproduction and reproductive processes [12]. Hu et al., identified 1683 DEGs (1136 up-regulated and 1045 down-regulated) in the ovaries of black Muscovy ducks at the early, peak and late egg-laying stages; they predicted that the *HOXA10, HtrA3, StAR, ZP2* and *TAT* genes were involved in regulating ovarian development at different laying stages, and several important pathways were found, including steroid hormone biosynthesis and ovarian steroidogenesis [13].

Ninghai indigenous chickens are commonly raised in southeastern Zhejiang Province, being a native chicken with high economic value for both meat and eggs. However, the poor egg-laying performance and short peak egg-laying period restrict the economic benefits of enterprises relating to this type of chicken. In this study, RNA-seq was used to identify differentially expressed genes in different egg-laying stages of Ninghai indigenous chickens. Through comprehensive analysis of the differential genes and pathways, in order to identify key pathways and candidate genes involved in the control of egg laying in Ninghai indigenous chickens, we aim to provide a basis for subsequent improvement of egg-laying performance, and also to help understand the molecular regulatory mechanisms of the egg-laying characteristics of chickens.

## 2. Materials and Methods

### 2.1. Animal and Sample Collection

Twelve Ninghai chickens (four 145-day-old chickens, four 204-day-old chickens and four 300-day-old chickens) were purchased from the Poultry Breeding Center of Ningbo Zhenning Animal Husbandry Co., Ltd. (Zhejiang, China) in Zhejiang Province. All the chickens were fed according to the same housing and feeding conditions. Ovarian tissues were collected from four randomly selected chickens at the 145-day-old, 204-day-old and 300-day-old, stages, respectively, and were immediately frozen in liquid nitrogen.

### 2.2. Ethical Statement

The animal study protocol followed the Chinese Animal Welfare Guidelines, and was approved by the Animal Welfare Committee of Zhejiang University (approval number: ZJU20190149).

### 2.3. RNA Sequencing (RNA-seq)

The collected hen ovarian tissues were delivered to the Novogene Bioinformatics Technology Co., Ltd. (Beijing, China), who conducted all the library preparation and sequencing. For the RNA sample preparations, a total of 3 g of RNA per sample was employed as the input material. Following the manufacturer’s instructions, sequencing libraries were created using the NEBNext^®^ UltraTM RNA Library Prep Kit for Illumina^®^ (NEB, California, USA), and index codes were added to attribute sequences to each sample. Briefly, mRNA was purified from total RNA using poly-T oligo-attached magnetic beads. Fragmentation was carried out using divalent cations under elevated temperature in NEBNext First Strand Synthesis Reaction Buffer(5X). First strand cDNA was synthesized using random hexamer primer and M-MuLV Reverse Transcriptase (RNase H). Second strand cDNA synthesis was subsequently performed using DNA Polymerase I and RNase H. Remaining overhangs were converted into blunt ends via exonuclease/polymerase activities. After adenylation of 3′ ends of DNA fragments, NEBNext Adaptor with hairpin loop structure was ligated to prepare for hybridization. In order to select cDNA fragments of preferentially 250~300 bp in length, the library fragments were purified with AMPure XP system (Beckman Coulter, Beverly, California, USA). Then, 3 µL USER Enzyme (NEB, California, USA) was used with size-selected, adaptor-ligated cDNA at 37 °C for 15 min followed by 5 min at 95 °C before PCR. Then, PCR was performed with Phusion High-Fidelity DNA polymerase, Universal PCR primers and Index (X) Primer. Finally, the PCR products were purified (AMPure XP system, California, USA) and the library quality was assessed on the Agilent Bioanalyzer 2100 system.

### 2.4. Transcriptomic Bioinformatics Analyses

In order to control the quality, in-house Perl scripts were used to process the raw data (raw readings) in the FASTQ format [14]. Clean data (clean reads) were acquired at this stage by eliminating adapter-containing reads, ploy-N containing reads, and low-quality reads from the raw data. The clean values for Q20, Q30, and GC content were computed at the same time [15]. All of the subsequent analyses were based on clean, high-quality data. The annotation data for the reference genome and gene models were acquired directly from the genome website (http://ftp.ensembl.org/pub/release-105/fasta/gallus_gallus/, http://ftp.ensembl.org/pub/release-105/gtf/gallus_gallus/, accessed on 10 June 2020). Hisat2 v2.0.5 was used to create a reference genome index, and Hisat2 v2.0.5 was used to align paired-end clean reads to the reference genome [16]. FeatureCounts v1.5.0-p3 was used to count the reads numbers mapped to each gene [17]. Then, the FPKM of each gene was calculated based on the length of the gene and the reads count mapped to this gene [18].

### 2.5. Differential Expression Analysis

The DESeq2 R package (1.16.1) was used to perform differential expression analyses on four biological replicates per condition [19]. Using a model based on the negative binomial distribution, DESeq2 provides statistical methods for identifying differential expression in digital gene expression data. The false discovery rate was controlled by adjusting the *p*-values using the Benjamini–Hochberg method [20]. Genes discovered by DESeq2 with an adjusted *p*-value of less than 0.05 were labeled as differentially expressed. The read counts for each sequenced library were modified using the edgeR software package through one scaling normalization factor prior to differential gene expression analysis. The edgeR R package (3.18.1) was used to perform differential expression analysis of two situations. The Benjamini–Hochberg method was used to alter the *p*-values. The threshold for substantially differential expression was established at a corrected P-value of 0.05 and an absolute fold-change of two.

### 2.6. GO and KEGG Enrichment Analysis of DEGs

The clusterProfiler R package was used to perform gene ontology (GO) enrichment analysis of differentially expressed genes, with gene length bias adjusted [21]. Differentially expressed genes substantially enriched GO keywords with a *p*-value of less than 0.05.

KEGG (http://www.genome.jp/kegg/, accessed on 15 August 2020) is a database resource for deducing high-level functions and utilities of biological systems, such as the cell, the organism, and the ecosystem, from molecular-level data, particularly large-scale molecular datasets generated by genome sequencing and other high-throughput experimental technologies [22]. To examine the statistical enrichment of differentially expressed genes in KEGG pathways, we utilized the clusterProfiler R program.

### 2.7. Gene Expression Analysis by qPCR

Using total RNA, 1 µg of total RNA was reverse transcribed to cDNA using the Prime Script RT kit (Takara, Hangzhou, China). qPCR was performed using SYBR Green PCR Master Mix (TaKaRa, HangZhou, China) on a StepOnePlus Real-Time PCR System. The 2^−ΔΔCt^ method was used to calculate the relative expression levels of genes [23], using β-actin as a control. Three biological replicates were used to analyze all mRNA expression. The primers used in the qPCR were designed using the NCBI website (https://www.ncbi.nlm.nih.gov/tools/primer-blast/, accessed on 11 March 2022) (Table S1).

## 3. Results

### 3.1. Sequencing Data and Read Mapping

A total of 701,634,762 clean reads were obtained from the 12 libraries with an average of 584,695,63.5 clean reads in each sample (the numbers of reads ranging from 51,709,354 to 66,121,522). The Q30 and GC content were 92.32% to 94.86% and 50.4% to 51.78%, respectively. More than 83.85% of the reads could be mapped to the chicken genome (NH145d, with 83.85% to 92.24% alignment; NH204d, with 88.69% to 91.12% alignment; and NH300d, with 88.64% to 91.52% alignment) and the unique map reads ranged from 82.17% to 90.94% (Table 1).

### 3.2. Differential Expression Genes

**Figure 1:**
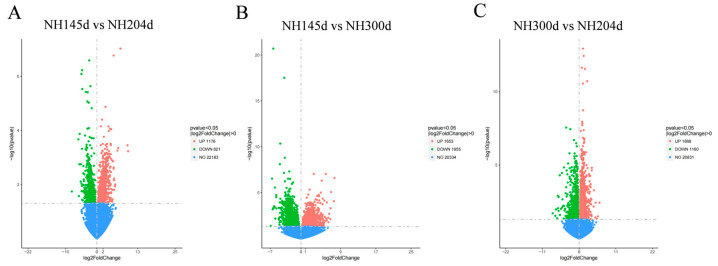
The volcano plot of DEGs in (**A**) NH145d vs. NH204d, (**B**) NH145d vs. NH300d, and (**C**) NH300d vs. NH204d. The *X*-axis represents the log2 fold change; the *Y*-axis represents the significance of differential expression *p* value on the–log10. Red dots: up-regulated DEGs; green dots: down-regulated DEGs; blue dots: non-DEGs.

**Figure 2:**
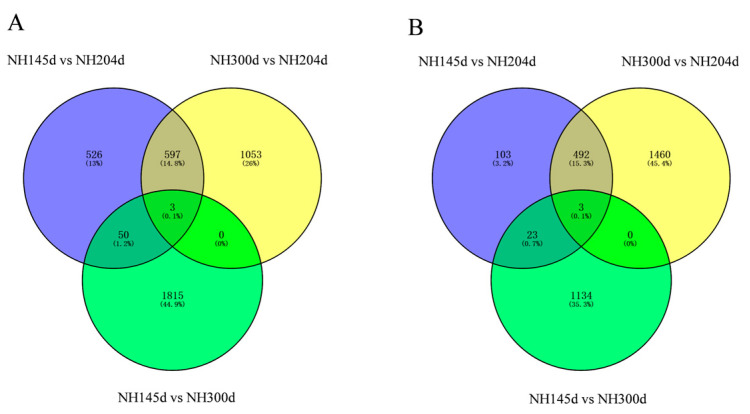
Venn diagram of DEGs of the three groups. (**A**) up-regulated DEGs; (**B**) down-regulated DEGs.

A total of 8433 DEGs were acquired from the three groups, with 1176 up-regulated genes and 621 down-regulated in NH145d vs. NH204d, 1653 up-regulated genes, and 1955 down-regulated in NH145d vs. NH300d, and 1868 up-regulated genes, and 1160 down-regulated in NH300d vs. NH204d. In addition, the two comparison groups included 73, 1004 and 1030 DEGs, respectively; and 65 genes were co-expressed among the three comparison groups (Figure 1 and Figure 2, Table S2).

### 3.3. GO and KEGG Analysis

**Figure 3:**
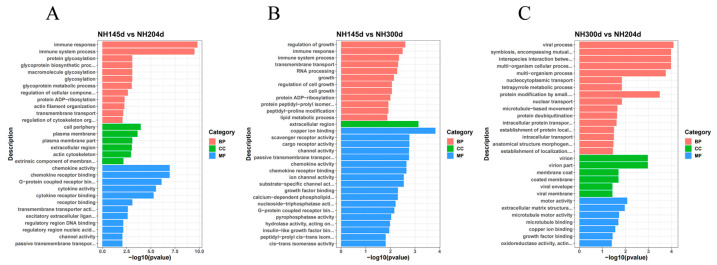
GO enrichment bar graph of the top 30 terms in (**A**) NH145d vs. NH204d, (**B**) NH145d vs. NH300d, (**C**) NH300d vs. NH204d. (BP: biological processes, CC: cellular components, CC: molecular functions).

DEGs from the three comparison groups were enriched for GO and KEGG analysis, in order to further elucidate the contribution of the specific signaling pathways in ovarian development. In GO analysis, the DEGs were annotated into three ontologies of the GO database: biological process (BP), cell component (CC), and molecular function (MF). In NH145d vs. NH204d, 3624 DEGs were annotated to 669 GO terms, covering 343 biological processes (BP), 78 cellular components (CC), and 248 molecular functions (MF). The items of evident enrichment included immune response, chemokine activity, chemokine receptor binding, cytokine activity, plasma membrane, and G-protein coupled receptor binding. In NH145d vs. NH300d, 7060 DEGs were assigned to 857 GO terms, including 448 BP, 118 CC, and 291 MF. The items of significant enrichment included the activity of passive transmembrane transporter activity, chemokine activity, chemokine receptor binding, and growth regulation. In NH300d vs. NH204d, 7244 DEGs were classified into 858 different GO terms, including 455 BP, 113 CC, and 290 MF. The GO terms included multi-organism cellular process, multi-organism process, and extracellular matrix structural constituent (Figure 3, Table S3).

**Figure 4:**
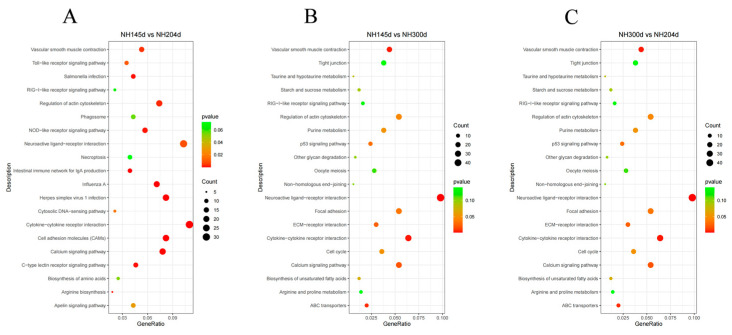
KEGG pathways for DEGs of the top 20 terms in (**A**) NH145d vs. NH204d, (**B**) NH145d vs. NH300d, (**C**) NH300d vs. NH204d.

For KEGG analysis, in NH45d vs. NH204d, 632 genes were enriched in 118 pathways, of which 16 pathways were significantly enriched, mainly in the cell adhesion molecules (CAMs), cytokine–cytokine receptor interaction, herpes simplex virus 1 infection, intestinal immune network for IgA, and the calcium signaling pathway. In NH145d vs. NH300d, 987 genes were enriched in 143 pathways, of which nine pathways were significantly enriched, mainly in the neuroactive ligand–receptor interaction, cytokine–cytokine receptor interaction, vascular smooth muscle contraction, and ABC transporters. In group NH300d vs. NH204d, 1140 genes were annotated in 148 pathways, of which four pathways were significantly enriched, namely progesterone-mediated oocyte maturation, RNA degradation, oocyte meiosis, and homologous recombination, respectively. Among the significantly enriched pathways, four pathways overlap in the two comparison groups NH145d vs. NH204d and NH145d vs. NH300d, namely cytokine–cytokine receptor interaction, the calcium signaling pathway, regulation of the actin cytoskeleton, vascular smooth muscle contraction, and neuroactive ligand–receptor interactions (Table 2 and Figure 4, Table S4).

**Figure 5:**
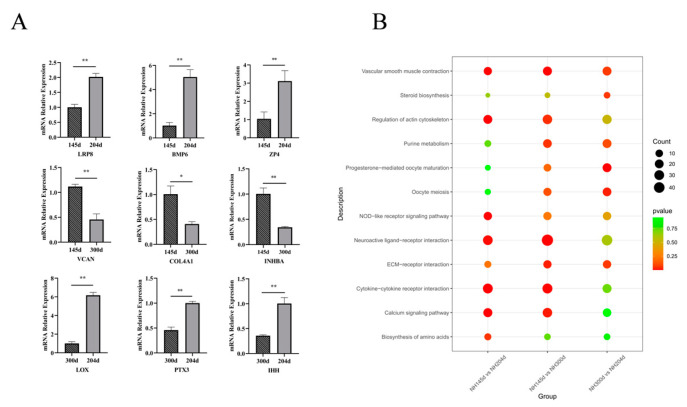
(**A**) Comparison of the expression of nine genes at different stages of egg production. Results are expressed as mean ± SD, * for *p* < 0.05 and ** for *p* <0.01. (**B**) The common KEGG pathway involved in egg production in the ovary in the three groups.

We performed RT-qPCR analysis of the expression of the nine genes at different times. As shown in Figure 5A, the qPCR expression results were consistent with the trend of RNA-seq results.

## 4. Discussion

Improving egg production is an important goal in poultry breeding. The ovary is a critical organ associated with poultry reproduction and is an essential tissue for finding candidate genes relating to egg production. By investigating ovarian gene expression patterns during development, we can contribute to improving egg production performance and to a better understanding of the reproductive physiology of poultry. In a comparative transcriptome analysis of ovarian tissues from high- and low-laying black and white Muscovy ducks, nine genes including *TGFβ2, NGFR, CEBPD, CPEB2, POSTN, SMOC1, FGF18, EFNA5* and *SDC4* were considered to be closely associated with egg production [24]. Transcriptome analysis of duck ovaries at different egg production rates revealed that genes such as *MC5R, APOD, ORAI1* and *DYRK4* were more active in the ovaries of high-laying ducks, and that the steroid biosynthetic pathway, calcium reabsorption pathways regulated by endocrine and other factors, circadian rhythms, neuroactive ligand–receptor interaction pathway, fatty acid biosynthesis, and calcium signaling pathways had more important roles in high-laying ducks [7]. Zhang et al.’s [25] comparative transcriptomic analysis of ovaries from high- and low-egg-laying Lingyun black-bone chickens found four genes, *FOXA2, MED37D, HSP70*, and *RXFP2*, and three signaling pathways that may be related to egg production, namely the longevity-regulating pathway, multiple species pathway, embryonic signaling pathway and PPAR signaling pathway.

### 4.1. Analysis of DEGs

The low-density lipoprotein (LDL) receptor-related protein 8 (*LRP8*) is a member of the LDL receptor family that has a role in endocytosis and signal transduction; *LRP8* paracrine interaction regulates follicular growth [26]. This gene is a component of the selenium delivery pathway to spermatogenic cells of mice [27], *LRP8* deficiency causes infertility in male mice, and it is necessary for sperm maturation [28]. This gene is also related to the development of the ovaries and follicles; it can affect the reproduction of female animals by regulating ovarian or follicular development [29,30]. In addition, this gene has also been reported to be a marker of fertility and follicle maturation [31]. In this study, *LRP8* was found to be significantly expressed at the peak of egg production, suggesting that LRP8 may have a positive role in regulating follicle growth in the ovary. The bone morphogenetic protein 6 (*BMP6*) is expressed in the ovary and is recognized as an autocrine/paracrine regulator of follicular and luteal cell proliferation and steroidogenesis [32]. *BMP6* is abundantly expressed in primordial, primary and secondary follicles [33]. During the transition from primordial to primary/secondary follicles, *BMP6* increases and enhances the growth of cultured secondary follicles [34]. In infertile women with endometriosis, the expression of the *BMP6* gene is relatively reduced [35]. A study found that *BMP6* regulates follicle dynamics and granulosa cell differentiation in the turkey ovary [36]. Zona pellucida glycoprotein 4 (*ZP4*) is a member of the zona pellucida (ZP) family, which is an extracellular glycoprotein matrix that surrounds oocytes [37]. Mutations in *ZP4* are associated with abnormal zona pellucida and with female infertility [38]. It also plays a vital role in fertilization by functioning as a “docking point” for spermatozoa binding, which is followed by the stimulation of the acrosome response in the zona-attached sperm [39]. In summary, *LRP8, BMP6*, and *ZP4* were significantly upregulated in the NH204d group compared to the NH145d group, suggesting that *LRP8, BMP6* and *ZP4* may have important regulatory roles in egg production mechanisms during peak egg production.

The versican (*VCAN*) protein is an extracellular matrix proteoglycan that stabilizes hyaluronan in the expanded extracellular matrix [40]. It has been found that the in vitro matured mouse mound-oocyte complex is deficient in *VCAN*, which may contribute to the poor health of in vitro matured oocytes and embryos [41]. *VCAN* expression has been reported as a marker of pregnancy [42]. Furthermore, Shen et al. [43] found the level of *VCAN* expression in cumulus cells to be positively correlated with the early embryo morphology score. The α1(IV) chain encoded by the *COL4A1* (type IV collagen α1 chain) gene is ubiquitously expressed and essential for the basement membrane’s stability [44]. It is strictly related to ovarian function and follicular development [45]. *COL4A1* has an essential role in regulating ECM remodeling during initial ovarian development in the bovine ovary and in regulating bovine fetal ovaries throughout pregnancy [46]. The inhibin β A subunit (*INHBA*) is a member of the transforming growth factor-β (TGFβ) superfamily, which encodes the βA-subunit of several activin and inhibin complexes [47]. *INHBA* regulates proliferation, apoptosis, and hormone synthesis in granulosa cells. Knocking out *INHBA* and inhibin subunit βB (*INHBB*) in granulosa cells impacts activin and inhibin production, resulting in sterile mice with a complex ovarian phenotype [48]. In the present study, *VCAN, COL4A1* and *INHBA* were upregulated in the NH145d group compared to the NH300d group, and the results suggest that the three genes may contribute to egg production performance in the early stages of laying, and may play a regulatory role in follicle development as well as ovarian matrix development.

Lysyl oxidase (*LOX*) activity catalyzes the final enzymatic reaction required in the biosynthesis of cross-linked mature collagens and elastin and, as such, is critical in the formation and deposition of the extracellular matrix (ECM) [49]. It has been reported that *LOX* is identified as part of the coordinated regulatory loop of the rat ovarian endocrine, paracrine and autocrine levels during follicular development [50]. Liu et al. highlighted the role of local cortisol in the amnion in downregulating *LOX* gene expression through a negative response element in the *LOX* promoter, with impaired *LOX* functions contributing to fetal membrane rupture [51]. Harlow et al. [52] found an essential role in regulating follicle development by *LOX* in the ovary. Pentraxin 3(*PTX3*) is a prototypic long pentraxin, a secreted protein member of the pentraxin family [53]. It plays a crucial role in organizing the cumulus oophorus extracellular matrix and in in vivo fertilization. Inactivating *PTX3* in mice can disrupt cumulus oophorus formation around the oocyte and reduce the fertilization rate. Zhang et al. [54] suggest that the gene expression in cumulus cells indicates pentraxin 3 as a possible marker for oocyte quality; Li et al. [55] found that microRNA-224 delays oocyte maturation through targeting *PTX3* in cumulus cells. Indian hedgehog (*IHH*), a member of the hedgehog (HH) family, plays a significant role in regulating numerous developmental processes, and is hormonally controlled and associated with co-maturation of the theca interna in the mammalian ovary [56,57]. Ovaries lacking the *IHH* show loss of the theca layer, blunted steroid production, impaired folliculogenesis, and failure to form corpora lutea [58]. *IHH* in epithelial cells generally acts as a paracrine growth factor for stromal cells in early gestation, and plays a role in peri-pregnancy preparation for uterine implantation [59]. *IHH* is also an essential mediator of progesterone signaling in the uterus, and expression of this factor is critical in mediating the communication between the uterine epithelium and stroma required for embryo implantation [60]. In this study, *LOX, PTX3*, and *IHH* were significantly increased in the NH204d group compared with the NH300d group, which suggests that the three genes are involved in the regulation of laying in the peak period, and may play an important role in ovarian hormonal regulation, promoting oocyte maturation.

### 4.2. GO and KEGG Analysis

GO (gene ontology) annotation and KEGG (Kyoto Encyclopedia of Genes and Genomes) analyses were used to further elucidate the biological roles of the DEGs. The GO results in the three comparison groups show that the biological processes and molecular functions in the ovary were allocated more DEGs than the cellular components. Chemokine activity and chemokine receptor binding were significantly enriched in NH145d vs. NH204d and NH145d vs. NH300d groups. Chemokines exert their effect by binding to their appropriate receptors, the expression levels of which may modulate their action [61]. The expression of chemokine and chemokine receptors in the endometrium suggests that autocrine and paracrine interactions involving these chemokines participate in endometrial physiology [62]. Chemokines are expressed in ovarian follicles and oocytes, potentially regulating follicular function and affecting reproductive lifespan [63]. The extracellular matrix is a fundamental structure present in all tissues of animal organisms. It exhibits various functions and is composed of many substances of different origins [64]. In addition, it has been shown that extracellular matrix components directly affect biological processes within the ovary, such as folliculogenesis, ovulation, and steroidogenesis [65]. Findings reported in the past few years indicate that the cumulus matrix plays a key role in the early events of in vivo fertilization [66]. Furthermore, the extracellular region and plasma membrane also play essential roles in the ovary; enrichment of the plasma membrane in the ovary is consistent with a recent report on the white Muscovy duck [67]. In a study of human sperm proteome profiles, GO analysis indicated that most of the differently identified sperm proteins were enriched in extracellular membrane-bounded organelles [68]. Moreover, “immune response” and “immune system process” were the biological processes with the highest DEGs, and they may play an essential role in the ovary of Ninghai chicken.

For KEGG, the signaling pathways, including cell adhesion molecules (CAMs), neuroactive ligand–receptor interaction, the calcium signaling pathway, and cytokine–cytokine receptor interaction, were the four most essential pathways associated with the ovary in Ninghai chickens. Cell adhesion is critical for ovary function and follicle development via interactions with the extracellular matrix and direct cell–cell contacts. The CAMs pathway has previously been shown to mediate a wide range of physiological functions, including cell-to-cell recognition, cell-to-matrix adhesion, and the development of early vertebrate embryos [69]. Furthermore, the calcium signaling pathway and neuroactive ligand–receptor interaction have been reported in more poultry ovaries; calcium (Ca^2+^) is a signaling molecule that regulates a wide range of biological functions [70], and the calcium signaling pathway plays an essential role in the egg production of ducks, while the pathway was also enriched in egg production in goose ovaries [71]. Ye et al. [72] found that the neuroactive ligand–receptor interaction pathway and the calcium signaling pathway were the key pathways controlling duck brooding. Another study also shows that this pathway affects goose egg production through ovarian metabolic function. In addition, the enrichment of the cytokine–cytokine receptor interaction pathway in the ovary in this study is consistent with the findings of Jinghai Yellow chicken, which also included the calcium signaling pathway and neuroactive ligand–receptor interaction [38]. In summary, several reports have shown the importance of cell adhesion molecules (CAMs), neuroactive ligand–receptor interaction, calcium signaling pathways, and cytokine–cytokine receptor interaction in chicken egg production [24,73]. In addition to these four pathways, we identified common KEGG pathways involved in reproduction during three periods (Figure 5B), including ECM-receptor interactions, steroid biosynthesis and progesterone-mediated oocyte maturation, three of which were found to be closely related to egg production. However, the other six pathways were rarely reported, and although not significantly enriched during all three periods, they may play an important role in poultry during egg production. In general, we have provided some new insights into the pathways associated with poultry egg production.

## 5. Conclusions

In this study, we identified 8433 differentially expressed genes through transcriptome sequencing analysis of the ovaries of Ninghai chickens at different egg-laying periods. We predicted nine genes involved in ovarian development—*LRP8, BMP6, ZP4, VCAN, COL4A1, INHBA, LOX, PTX3*, and *IHH*—through the calcium pathway and neurotransmitter receptor interaction pathways. The results of this study will help in the investigation of the regulatory network and molecular mechanisms of ovarian development in Ninghai chickens, and will provide valuable information for understanding the mechanisms of ovarian action and the molecular approaches towards improving egg production performance in chickens.

## Figures and Tables

**Table 1:** Sequencing data.

Sample	Raw Reads	Clean Reads	Total Map	Q20	Q30	GC Pct
NH300d_1	66888644	66121522	60514494 (91.52%)	97.88%	94.22%	50.69%
NH300d_2	63552028	62595376	55486806 (88.64%)	97.66%	93.86%	51.78%
NH300d_3	58809960	58226874	54031796 (92.8%)	98.01%	94.49%	50.84%
NH300d_4	52437050	51709354	47297226 (91.47%)	98%	94.52%	50.4%
NH204d_1	59434470	58593266	53253424 (90.89%)	97.86%	94.22%	50.91%
NH204d_2	53435540	52503528	47500358 (90.47%)	97.85%	94.22%	50.53%
NH204d_3	62255842	61476260	56020220 (91.12%)	97.82%	94.17%	51.38%
NH204d_4	53250580	52498894	46560294 (88.69%)	97.85%	94.17%	51.69%
NH145d_1	73572594	72533526	64007420 (88.25%)	97.82%	94.2%	51.65%
NH145d_2	57911030	56397798	47287950 (83.85%)	96.71%	92.32%	50.4%
NH145d_3	57453868	56745306	51098056 (90.05%)	97.95%	94.46%	51.52%
NH145d_4	52929654	52233058	48179156 (92.24%)	97.81%	94.02%	50.81%

**Table 2:** The significant enrichment pathways of KEGG.

Group	Pathway	GeneRatio	BgRatio	*p*	Q	KEGG ID
NH145dvsNH204d	Cell adhesion molecules (CAMs)	23/282	107/4684	5.1 × 10^−8^	6.0 × 10^−6^	gga04514
	Cytokine–cytokine receptor interaction	31/282	187/4684	1.4 × 10^−7^	8.5 × 10^−6^	gga04060
	Herpes simplex virus 1 infection	23/282	132/4684	2.7 × 10^−6^	1.1 × 10^−4^	gga05168
	Intestinal immune network for IgA production	11/282	36/4684	4.8 × 10^−6^	1.4 ×10^−4^	gga04672
	Influenza A	20/282	112/4684	8.4 × 10^−6^	2.0 ×10^−4^	gga05164
	Calcium signaling pathway	22/282	168/4684	4.0 × 10^−4^	7.8 ×10^−3^	gga04020
	C–type lectin receptor signaling pathway	13/282	80/4684	8.5 × 10^−4^	1.3 ×10^−2^	gga04625
	Salmonella infection	12/282	71/4684	9.3 × 10^−4^	1.3 ×10^−2^	gga05132
	Arginine biosynthesis	5/282	14/4684	9.7 × 10^−4^	1.3 ×10^−2^	gga00220
	NOD–like receptor signaling pathway	16/282	115/4684	1.3 × 10^−3^	1.5 ×10^−2^	gga04621
	Regulation of actin cytoskeleton	21/282	182/4684	2.7 × 10^−3^	3.0 ×10^−2^	gga04810
	Vascular smooth muscle contraction	15/282	116/4684	3.7 × 10^−3^	3.7 ×10^−2^	gga04270
	Neuroactive ligand–receptor interaction	29/282	305/4684	8.5 × 10^−3^	7.6 ×10^−2^	gga04080
	Toll-like receptor signaling pathway	10/282	72/4684	1.0 × 10^−2^	8.7 ×10^−2^	gga04620
	Cytosolic DNA-sensing pathway	6/282	35/4684	1.7 × 10^−2^	1.3 ×10^−1^	gga04623
	Apelin signaling pathway	12/282	109/4684	3.0 × 10^−2^	2.2 ×10^−1^	gga04371
NH145dvsNH300d	Neuroactive ligand–receptor interaction	49/499	305/4685	1.7 × 10^−3^	2.3 ×10^−1^	gga04080
	Cytokine–cytokine receptor interaction	32/499	187/4685	4.1 × 10^−3^	2.3 ×10^−1^	gga04060
	Vascular smooth muscle contraction	22/499	116/4685	4.7 × 10^−3^	2.3 ×10^−1^	gga04270
	ABC transporters	10/499	40/4685	7.6 × 10^−3^	2.7 ×10^−1^	gga02010
	Calcium signaling pathway	27/499	169/4685	1.9 × 10^−2^	5.5 ×10^−1^	gga04020
	ECM–receptor interaction	15/499	82/4685	2.4 × 10^−2^	5.8 ×10^−1^	gga04512
	p53 signaling pathway	12/499	63/4685	3.1 × 10^−2^	6.3 ×10^−1^	gga04115
	Focal adhesion	27/499	178/4685	3.5 × 10^−2^	6.3 ×10^−1^	gga04510
	Regulation of actin cytoskeleton	27/499	182/4685	4.5 × 10^−2^	6.8 ×10^−1^	gga04810
NH300dvsNH204d	Progesterone–mediated oocyte maturation	21/611	76/4684	5.0 × 10^−4^	7.3 ×10^−2^	gga04914
	RNA degradation	18/611	67/4684	1.7 × 10^−3^	1.3 ×10^−1^	gga03018
	Oocyte meiosis	19/611	92/4684	2.6 × 10^−2^	1.0	gga04114
	Homologous recombination	9/611	37/4684	4.4 × 10^−2^	1.0	gga03440

## Data Availability

The sequence data have been submitted to the NCBI SRA database under accession number PRJNA807504 and PRJNA798791.

## Supplementary Materials

The following supporting information can be downloaded at: https://www.mdpi.com/article/10.3390/genes13040595/s1. Table S1: List of primer sequences used for the qRT-PCR experiments. Table S2: List of all DEGs of the three comparison groups. Table S3: List of GO terms of the three comparison groups. Table S4: List of KEGG pathways of the three comparison groups.

## References

[1] Zaheer K. (2015). An updated review on chicken eggs: Production, consumption, management aspects and nutritional benefits to human health. Food Sci. Nutr..

[2] Iannotti L.L., Lutter C.K., Bunn D.A., Stewart C.P. (2014). Eggs: The uncracked potential for improving maternal and young child nutrition among the world’s poor. Nutr. Rev..

[3] Sun X., Chen X., Zhao J., Ma C., Yan C., Liswaniso S., Xu R., Qin N. (2021). Transcriptome comparative analysis of ovarian follicles reveals the key genes and signaling pathways implicated in hen egg production. BMC Genom..

[4] Webb R., Garnsworthy P.C., Gong J.G., Armstrong D.G. (2004). Control of follicular growth: Local interactions and nutritional influences. J. Anim. Sci..

[5] Mishra S.K., Chen B., Zhu Q., Xu Z., Ning C., Yin H., Wang Y., Zhao X., Fan X., Yang M. (2020). Transcriptome analysis reveals differentially expressed genes associated with high rates of egg production in chicken hypothalamic-pituitary-ovarian axis. Sci. Rep..

[6] Woodruff T.K., Shea L.D.J.R.S. (2007). The role of the extracellular matrix in ovarian follicle development. Reprod. Sci..

[7] Tao Z., Song W., Zhu C., Xu W., Liu H., Zhang S., Huifang L. (2017). Comparative transcriptomic analysis of high and low egg-producing duck ovaries. Poult. Sci..

[8] Zhang X., Huang L., Wu T., Feng Y., Ding Y., Ye P., Yin Z. (2015). Transcriptomic analysis of ovaries from pigs with high and low litter size. PLoS ONE.

[9] Lan D., Xiong X., Huang C., Mipam T.D., Li J. (2016). Toward understanding the genetic basis of yak ovary reproduction: A characterization and comparative analyses of estrus ovary transcriptiome in yak and cattle. PLoS ONE.

[10] Miao X., Luo Q. (2013). Genome-wide transcriptome analysis between small-tail Han sheep and the Surabaya fur sheep using high-throughput RNA sequencing. Reproduction.

[11] Miao X., Luo Q., Qin X. (2016). Genome-wide transcriptome analysis in the ovaries of two goats identifies differentially expressed genes related to fecundity. Gene.

[12] Zou K., Asiamah C.A., Lu L.-L., Liu Y., Pan Y., Chen T., Zhao Z., Su Y. (2020). Ovarian transcriptomic analysis and follicular development of Leizhou black duck. Poult. Sci..

[13] Hu Z., Liu J., Cao J., Zhang H., Liu X. (2021). Ovarian transcriptomic analysis of black Muscovy duck at the early, peak and late egg-laying stages. Gene.

[14] Andrews S. (2010). FastQC: A quality control tool for high throughput sequence data. Babraham Bioinformatics.

[15] Trapnell C., Pachter L., Salzberg S.L. (2009). TopHat: Discovering splice junctions with RNA-Seq. Bioinformatics.

[16] Kim D., Landmead B., Salzberg S.L. (2015). HISAT: A fast spliced aligner with low memory requirements. Nat. Methods.

[17] Liao Y., Smyth G.K., Shi W. (2014). FeatureCounts: An efficient general purpose program for assigning sequence reads to genomic features. Bioinformatics.

[18] Garber M., Grabherr M.G., Guttman M., Trapnell C. (2011). Computational methods for transcriptome annotation and quantification using RNA-seq. Nat. Methods.

[19] Love M.I., Huber W., Anders S. (2014). Moderated estimation of fold change and dispersion for RNA-seq data with DESeq2. Genome Biol..

[20] Benjamini Y., Hochberg Y. (1995). Controlling the False Discovery Rate - a Practical and Powerful Approach To Multiple Testing. J R Stat. Soc. B.

[21] Ashburner M., Ball C.A., Blake J.A., Botstein D., Butler H., Cherry J.M., Davis A.P., Dolinski K., Dwight S.S., Eppig J.T. (2000). Gene Ontology: Tool for the unification of biology. Nat. Genet..

[22] Kanehisa M., Goto S. (2000). KEGG: Kyoto Encyclopedia of Genes and Genomes. Nucleic Acids Res..

[23] Livak K.J., Schmittgen T.D. (2001). Analysis of relative gene expression data using real-time quantitative PCR and the 2− ΔΔCT method. Methods.

[24] Bao X.Y., Song Y.P., Li T., Zhang S.S., Huang L.H., Zhang S.Y., Cao J.T., Liu X.L., Zhang J.Q. (2021). Comparative Transcriptome Profiling of Ovary Tissue between Black Muscovy Duck and White Muscovy Duck with High- and Low-Egg Production. Genes.

[25] Zhang Q.Y., Wang P.F., Cong G.L., Liu M.H., Shi S.R., Shao D., Tan B.J. (2021). Comparative transcriptomic analysis of ovaries from high and low egg-laying Lingyun black-bone chickens. Vet. Med. Sci..

[26] Akhavan S.R., Falahatkar B., McCormick S.P., Lokman P.M., Physiology C. (2020). Changes in lipid biology during ovarian development in farmed beluga sturgeon, *Huso huso* L.. Am. J. Physiol.-Regul. Integr. Comp. Physiol..

[27] Olson G.E., Winfrey V.P., NagDas S.K., Hill K.E., Burk R.F. (2007). Apolipoprotein E receptor-2 (ApoER2) mediates selenium uptake from selenoprotein P by the mouse testis. J. Biol. Chem..

[28] Andersen O.M., Yeung C.-H., Vorum H., Wellner M., Andreassen T.K., Erdmann B., Mueller E.-C., Herz J., Otto A., Cooper T.G. (2003). Essential role of the apolipoprotein E receptor-2 in sperm development. J. Biol. Chem..

[29] Yao J., Chen Z., Xu G., Wang X., Ning Z., Zheng J., Qu L., Yang N. (2010). Low-density lipoprotein receptor-related protein 8 gene association with egg traits in dwarf chickens. Poult. Sci..

[30] Wang C., Li S., Li C., Yu G., Feng Y., Peng X., Gong Y.J.B.P.S. (2013). Molecular cloning, expression and association study with reproductive traits of the duck LRP8 gene. Br. Poult. Sci..

[31] Dias F., Khan M., Sirard M., Adams G., Singh J. (2018). Transcriptome analysis of granulosa cells after conventional vs long FSH-induced superstimulation in cattle. BMC Genom..

[32] Chang H.-M., Qiao J., Leung P.C. (2017). Oocyte–somatic cell interactions in the human ovary—novel role of bone morphogenetic proteins and growth differentiation factors. Hum. Reprod. Update.

[33] Zhu G., Guo B., Pan D., Mu Y., Feng S. (2008). Expression of bone morphogenetic proteins and receptors in porcine cumulus–oocyte complexes during in vitro maturation. Anim. Reprod. Sci..

[34] Frota I.M., Leitão C.C., Costa J.J., van den Hurk R., Saraiva M.V., Figueiredo J.R., Silva J.R. (2013). Levels of BMP-6 mRNA in goat ovarian follicles and in vitro effects of BMP-6 on secondary follicle development. Zygote.

[35] De Conto E., Matte U., Cunha-Filho J.S. (2021). BMP-6 and SMAD4 gene expression is altered in cumulus cells from women with endometriosis-associated infertility. Acta Obstet. Gynecol. Scand..

[36] Ghanem K., Johnson A.L. (2018). Follicle dynamics and granulosa cell differentiation in the turkey hen ovary. Poult. Sci..

[37] Izquierdo-Rico M., Jimenez-Movilla M., Llop E., Perez-Oliva A., Ballesta J., Gutierrez-Gallego R., Jimenez-Cervantes C., Aviles M. (2009). Hamster zona pellucida is formed by four glycoproteins: ZP1, ZP2, ZP3, and ZP4. Proteome Res..

[38] Zhou Z., Ni C.X., Wu L., Chen B.B., Xu Y., Zhang Z.H., Mu J., Li B., Yan Z., Fu J. (2019). Novel mutations in ZP1, ZP2, and ZP3 cause female infertility due to abnormal zona pellucida formation. Hum. Genet..

[39] Serizawa M., Kinoshita M., Rodler D., Tsukada A., Ono H., Yoshimura T., Kansaku N., Sasanami T. (2011). Oocytic expression of zona pellucida protein ZP4 in Japanese quail (Coturnix japonica). Anim. Sci. J..

[40] Russell D.L., Ochsner S.A., Hsieh M., Mulders S., Richards J.S. (2003). Hormone-regulated expression and localization of versican in the rodent ovary. Endocrinology.

[41] Dunning K.R., Lane M., Brown H.M., Yeo C., Robker R.L., Russell D.L. (2007). Altered composition of the cumulus-oocyte complex matrix during in vitro maturation of oocytes. Hum. Reprod..

[42] Papler T.B., Bokal E.V., Maver A., Lovrečić L. (2015). Specific gene expression differences in cumulus cells as potential biomarkers of pregnancy. Reprod. Biomed. Online.

[43] Shen Q., Chen M., Zhao X., Liu Y., Ren X., Zhang L. (2020). Versican expression level in cumulus cells is associated with human oocyte developmental competence. Syst. Biol. Reprod. Med..

[44] Terauchi K.J., Miyagawa S., Iguchi T., Sato T. (2020). Hedgehog signaling regulates the basement membrane remodeling during folliculogenesis in the neonatal mouse ovary. Cell Tissue Res..

[45] Franchi F.F., Hernandes M.P., Ferreira A.L.C., de Lima V.A.V., de Oliveira Mendes L., de Aquino A.M., Scarano W.R., de Souza Castilho A.C. (2020). Fractal analysis and histomolecular phenotyping provides insights into extracellular matrix remodeling in the developing bovine fetal ovary. Biochem. Biophys. Res. Commun..

[46] Tang J., Hu W., Chen S., Di R., Liu Q., Wang X., He X., Gan S., Zhang X., Zhang J. (2019). The genetic mechanism of high prolificacy in small tail han sheep by comparative proteomics of ovaries in the follicular and luteal stages. J. Proteom..

[47] Bao Y., Yao X., Li X., Ei-Samahy M., Yang H., Liang Y., Liu Z., Wang F. (2021). INHBA transfection regulates proliferation, apoptosis and hormone synthesis in sheep granulosa cells. Theriogenology.

[48] Pangas S.A., Jorgez C.J., Tran M., Agno J., Li X.H., Brown C.W., Kumar T.R., Matzuk M.M. (2007). Intraovarian activins are required for female fertility. Mol. Endocrinol..

[49] Wei S.Z., Gao L., Wu C.J., Qin F., Yuan J.H. (2020). Role of the lysyl oxidase family in organ development (Review). Exp. Ther. Med..

[50] Laczko R., Csiszar K.J.B. (2020). Lysyl oxidase (LOX): Functional contributions to signaling pathways. Biomolecules.

[51] Liu C., Zhu P., Wang W., Li W., Shu Q., Chen Z.-J., Myatt L., Sun K. (2016). Inhibition of lysyl oxidase by prostaglandin E2 via EP2/EP4 receptors in human amnion fibroblasts: Implications for parturition. Mol. Cell. Endocrinol..

[52] Harlow C.R., Rae M., Davidson L., Trackman P.C., Hillier S.G. (2003). Lysyl oxidase gene expression and enzyme activity in the rat ovary: Regulation by follicle-stimulating hormone, androgen, and transforming growth factor-β superfamily members in vitro. Endocrinology.

[53] Irez T., Sahin Y., Malik E., Guralp O. (2022). The Predictive Value of the Pentraxin 3 Concentration in Cumulus Cell Culture Media for the Embryo Implantation. J. Gynecol. Obstet. Hum. Reprod..

[54] Zhang X., Jafari N., Barnes R.B., Confino E., Milad M., Kazer R.R. (2005). Studies of gene expression in human cumulus cells indicate pentraxin 3 as a possible marker for oocyte quality. Fertil. Steril..

[55] Li X., Wang H., Sheng Y., Wang Z. (2017). MicroRNA-224 delays oocyte maturation through targeting Ptx3 in cumulus cells. Mech. Dev..

[56] Hatzirodos N., Irving-Rodgers H.F., Hummitzsch K., Harland M.L., Morris S.E., Rodgers R.J. (2014). Transcriptome profiling of granulosa cells of bovine ovarian follicles during growth from small to large antral sizes. BMC Genom..

[57] Liu C., Peng J., Matzuk M.M., Yao H.H.-C. (2015). Lineage specification of ovarian theca cells requires multicellular interactions via oocyte and granulosa cells. Nat. Commun..

[58] Matsumoto H., Zhao X., Das S.K., Hogan B.L., Dey S.K. (2002). Indian hedgehog as a progesterone-responsive factor mediating epithelial–mesenchymal interactions in the mouse uterus. Dev. Biol..

[59] Bhurke A.S., Bagchi I.C., Bagchi M.K. (2016). Progesterone-Regulated Endometrial Factors Controlling Implantation. Am. J. Reprod. Immunol..

[60] Lee K., Jeong J., Kwak I., Yu C.-T., Lanske B., Soegiarto D.W., Toftgard R., Tsai M.-J., Tsai S., Lydon J.P. (2006). Indian hedgehog is a major mediator of progesterone signaling in the mouse uterus. Nature Genet..

[61] Mulayim N., Palter S.F., Kayisli U.A., Senturk L., Arici A. (2003). Chemokine receptor expression in human endometrium. Biol. Reprod..

[62] Zhang J., Lathbury L.J., Salamonsen L.A. (2000). Expression of the chemokine eotaxin and its receptor, CCR3, in human endometrium. Biol. Reprod..

[63] Santos A.G.A., Pereira L.A.A.C., Viana J.H.M., Russo R.C., Campos-Junior P.H.A. (2020). The CC-chemokine receptor 2 is involved in the control of ovarian folliculogenesis and fertility lifespan in mice. J. Reprod. Immunol..

[64] Walma D.A.C., Yamada K.M. (2020). The extracellular matrix in development. Development.

[65] Kulus J., Kulus M., Kranc W., Jopek K., Zdun M., Józkowiak M., Jaśkowski J.M., Piotrowska-Kempisty H., Bukowska D., Antosik P. (2021). Transcriptomic Profile of New Gene Markers Encoding Proteins Responsible for Structure of Porcine Ovarian Granulosa Cells. Biol.-Basel.

[66] Zhuo L., Yoneda M., Zhao M., Yingsung W., Yoshida N., Kitagawa Y., Kawamura K., Suzuki T., Kimata K.J.J.O.B.C. (2001). Defect in SHAP-hyaluronan complex causes severe female infertility: A study by inactivation of the bikunin gene in mice. J. Biol. Chem..

[67] Bello S.F., Xu H., Guo L., Li K., Zheng M., Xu Y., Zhang S., Bekele E.J., Bahareldin A.A., Zhu W. (2021). Hypothalamic and ovarian transcriptome profiling reveals potential candidate genes in low and high egg production of white Muscovy ducks (Cairina moschata). Poult. Sci..

[68] Li S., Ao L., Yan Y., Jiang J., Chen B., Duan Y., Shen F., Chen J., Inglis B., Ni R. (2019). Differential motility parameters and identification of proteomic profiles of human sperm cryopreserved with cryostraw and cryovial. Clin. Proteom..

[69] Heffner K., Hizal D.B., Majewska N.I., Kumar S., Dhara V.G., Zhu J., Bowen M., Hatton D., Yerganian G., Yerganian A.J.S.R. (2020). Expanded Chinese hamster organ and cell line proteomics profiling reveals tissue-specific functionalities. Sci. Rep..

[70] Park Y.-J., Yoo S.-A., Kim M., Kim W.-U. (2020). The Role of Calcium–Calcineurin–NFAT Signaling Pathway in Health and Autoimmune Diseases. Front. Immunol..

[71] Wu Y., Zhao X., Chen L., Wang J., Duan Y., Li H., Lu L. (2020). Transcriptomic analyses of the hypothalamic-pituitary-gonadal axis identify candidate genes related to egg production in Xinjiang Yili geese. Animals.

[72] Ye P., Ge K., Li M., Yang L., Jin S., Zhang C., Chen X., Geng Z. (2019). Egg-laying and brooding stage-specific hormonal response and transcriptional regulation in pituitary of Muscovy duck (Cairina moschata). Poult. Sci..

[73] Chen X., Sun X., Chimbaka I.M., Qin N., Xu X., Liswaniso S., Xu R., Gonzalez J.M. (2021). Transcriptome analysis of ovarian follicles reveals potential pivotal genes associated with increased and decreased rates of chicken egg production. Front. Genet..

